# Metabolic cost of osmoregulation in a hypertonic environment in the invasive African clawed frog *Xenopus laevis*

**DOI:** 10.1242/bio.016543

**Published:** 2016-06-22

**Authors:** Isaac Peña-Villalobos, Cristóbal Narváez, Pablo Sabat

**Affiliations:** 1 Departamento de Ciencias Ecológicas, Facultad de Ciencias, Universidad de Chile, Santiago 7800003, Chile; 2 Center of Applied Ecology and Sustainability (CAPES), Pontificia Universidad Católica de Chile, Santiago 6513677, Chile

**Keywords:** Metabolic enzymes, Osmoregulation, Salinity, Standard metabolic rate (SMR), *Xenopus laevis*

## Abstract

Studies of aquatic invertebrates reveal that salinity affects feeding and growth rates, reproduction, survival, and diversity. Little is known, however, about how salinity impacts the energy budget of vertebrates and amphibians in particular. The few studies focused on this topic in vertebrates suggest that the ingestion of salts and the resulting osmoregulatory activity is energetically expensive. We analyzed the effect of saline acclimation on standard metabolic rates (SMR) and the activities of metabolic enzymes of internal organs and osmoregulatory variables (plasma osmolality and urea plasma level) in females of *Xenopus laevis* by means of acclimating individuals to an isosmotic (235 mOsm NaCl; ISO group) and hyper-osmotic (340 mOsm NaCl; HYP group) environment for 40 days. After acclimation, we found that total and mass-specific SMR was approximately 80% higher in the HYP group than those found in the ISO group. These changes were accompanied by higher citrate synthase activities in liver and heart in the HYP group than in the ISO group. Furthermore, we found a significant and positive correlation between metabolic rates and plasma urea, and citrate synthase activity in liver and heart. These results support the notion that the cost of osmoregulation is probably common in most animal species and suggest the existence of a functional association between metabolic rates and the adjustments in osmoregulatory physiology, such as blood distribution and urea synthesis.

## Abstract (precis)

**Summary:** A significant increase in energy expenditure of *Xenopus laevis* exposed to hyperosmotic conditions is associated with urea concentration and a long-term effect of increasing the metabolic enzyme activities of liver and heart.

## INTRODUCTION

Increasing salinity of watercourses is known as secondary or anthropogenic salinization and constitutes an acute form of environmental disturbance (Mack et al., 2000; Smith et al., 2007). Several studies reported the effect of salinity at different levels of biological organization such as feeding and growth rates, reproduction, survival, and species diversity, especially in aquatic invertebrates (Aladin, 1991; Frey, 1993; Jeppesen et al., 1994; Achuthankutty et al., 2000; Schallenberg et al., 2003; Amsinck et al., 2005; Sarma et al., 2006; Heine-Fuster et al., 2010; Karraker and Gibbs, 2011). Thus, living in salty environments is considered a challenge for animals because maintenance of constant ionic and osmotic conditions of internal fluids is necessary for the adequate function of enzymatic reactions and its dependent processes (Bentley, 1970; Evans, 2009).

Such homeostatic challenge is of particular significance in those organisms lacking behavioral or physiological mechanisms to avoid the impact of salinity (e.g. the intake of ions and dehydration) as in vertebrate intertidal species (Janssen et al., 2015), and in particular for amphibians, especially species that spend all their life in water or depend on fresh water supplies for reproduction (see Hsu et al., 2012). Recent studies have analyzed the ecological effects of salinity on amphibian species, such as changes on survival, larval growth, metamorphosis and morphological alterations of gill epithelium (Christy and Dickman, 2002; Karraker et al., 2010; Karraker and Gibbs, 2011; Hsu et al., 2012; Bernabò et al., 2013; Woolrich-Piña et al., 2015). Overall, those studies reported negative effects of the increase of salinity in biological performance in amphibians. In this vein, it has been noted that amphibians have a poor tolerance to salt water (Darwin, 1859; Bentley, 1970; Jørgensen, 1997) due the lack of ability to concentrate urine to eliminate greater amounts of excess electrolites through the kidney (Gordon, 1962; Jard and Morel, 1963; McClanahan, 1967; Bentley, 1970), and their limited ability to remain hyperosmotic to the external solution (Katz, 1989).

Despite these limitations, seminal studies have reported the presence of particular mechanisms of osmoregulation in amphibians living in environments with variable salinity (Krogh, 1937; Krogh, 1939).

In amphibians, the skin plays a major role in ion and water exchanges (Shoemaker and Nagy, 1977). The skin of anurans is capable of net inward transport of Na^+^ and Cl^−^, even when the animals are exposed to very diluted water (Boutilier et al., 1992); although in hyperosmotic conditions, some anurans can decrease water permeability through the skin (Dicker and Elliott, 1970; Katz and Weisberg, 1971). When toads and frogs are acclimated to hyper-saline solutions the skin develops a transport capacity of urea into the animal (Katz and Hanke, 1993), thus the synthesis and/or accumulation of organic and inorganic osmolytes raise internal osmotic pressure, thereby reducing the transcutaneous gradient for water loss (Shoemaker, 1964; Katz and Hoffman, 1990; Katz and Hanke, 1993). For example, *Xenopus laevis* is ammonotelic under freshwater conditions and becomes ureotelic in hyperosmotic conditions and aestivation (Schlisio et al., 1975; Schröck and Hanke, 1979; Lindley et al., 2007). This last condition is attained by the increased synthesis and accumulation of urea, accompanied by a twofold increase in the activity of the ornithine-urea cycle enzymes present in liver (McBean and Goldstein, 1967). Accordingly, Jørgensen (1997) reported that the anurans *Scaphiopus* spp., *Bufo viridis*, *Xenopus laevis* and *Rana cancrivora*, and the urodeles *Ambystoma tigrinum* and *Batrachoseps* spp., are able to synthesize urea in order to maintain a positive water balance in hyperosmotic conditions. Thus, long-term acclimated amphibians to saline environments remain slightly hyperosmotic by means of storage of urea and other non-electrolytes, increasing salinity tolerance.

Furthermore, the intake of salts through the food, drinking, or across the body surface by animals represents a significant energetic cost, suggesting that maintaining an active osmoregulatory machinery is energetically expensive and could partially explain diet and/or habitat selection (Hoar and Randall, 1969; Nehls, 1996; Tseng and Hwang, 2008; Evans, 2009; Gutiérrez et al., 2011). In this vein, some studies revealed that up to 50% of the total fish energy budget is allocated to osmoregulation (see Boeuf and Payan, 2001). For example, the euryhaline *Paralichthys orbignyanus* exhibits a lower growth rate in fresh water, suggesting higher energy expenditure associated to a branchial Na^+^, K^+^-ATPase activity (Sampaio and Bianchini, 2002). Furthermore, ureotelic regulation through urea accumulation in plasma and tissues by cartilaginous fishes and some anurans seems to be an energetically expensive strategy, involving both urea synthesis via the ornithine cycle (Boeuf and Payan, 2001; LeMoine and Walsh, 2015) and the recovery of urea by active tubular transport (see Uchiyama and Konno, 2006). For example, the oxygen consumption of the obligated ureotelic tilapia (*Alcolapia graham*) acclimated to dilute water decreased by 40–68% along with urea levels compared to those acclimated to saline water (Wood et al., 2002), inhabiting a hyperosmotic environment in several species of aquatic animals and also in some non-aquatic species (Gutiérrez et al., 2011; Peña-Villalobos et al., 2013). To the best of our knowledge, no previous study has evaluated the putative metabolic cost of inhabiting hyperosmotic environments in amphibians.

In this paper we experimentally evaluate the effect of environmental salinity on standard rates of energy expenditure, along with the plasma osmolality, urea plasma level, and activities of metabolic enzymes of internal organs in the African clawed frog *Xenopus laevis* (Daudin, Pipidae: Anura). This amphibian species is a relatively efficient hyperosmoregulator, with a maximum tolerance of 400 mOsm of NaCl (Katz and Hanke, 1993). In the pre-advent of modern agriculture this species occurred at low densities in most watercourses, however now there have been multiple sites recognized where this species has successfully invaded (see Tinsley and McCoid, 1996; Measey, 1998; Lobos and Measey, 2002), including a breeding population in brackish water (salinity value not recorded) in California (Munsey, 1972). This species is a well-suited model to study the cost associated with osmoregulation under various conditions because it is cosmopolitan in having some osmoregulatory capacity. We hypothesize that *X. laevis* acclimated to a hyperosmotic medium will exhibit: (i) higher resting metabolic rates, (ii) higher masses and metabolic enzymatic activities of heart and liver, and (iii) higher urea plasma levels than those acclimated to isosmotic conditions.

## RESULTS

### SMR and blood parameters

Body mass was not affected by experimental treatment, however groups lost around 10% of their body mass during the acclimation period (see Table 1 for values and statistics). Total SMR (ml O_2_ h^−1^) was higher in the HYP group than in the ISO group (173%; Table 1). We observed a significant effect of the treatment in the residuals of total SMR, with a higher value in the HYP group than in the ISO group (*F*_(1,10)_=6.533, *P*=0.029; Pt: *P*=0.013). We also found a positive association between the residues of total metabolic rate and residues of the intestinal mass (r^2^=0.449; Pt: *P*=0.002) and the mass of fat bodies (r^2^=0.548, *P*=0.006; Pt: *P*=0.018).

**Table 1.**
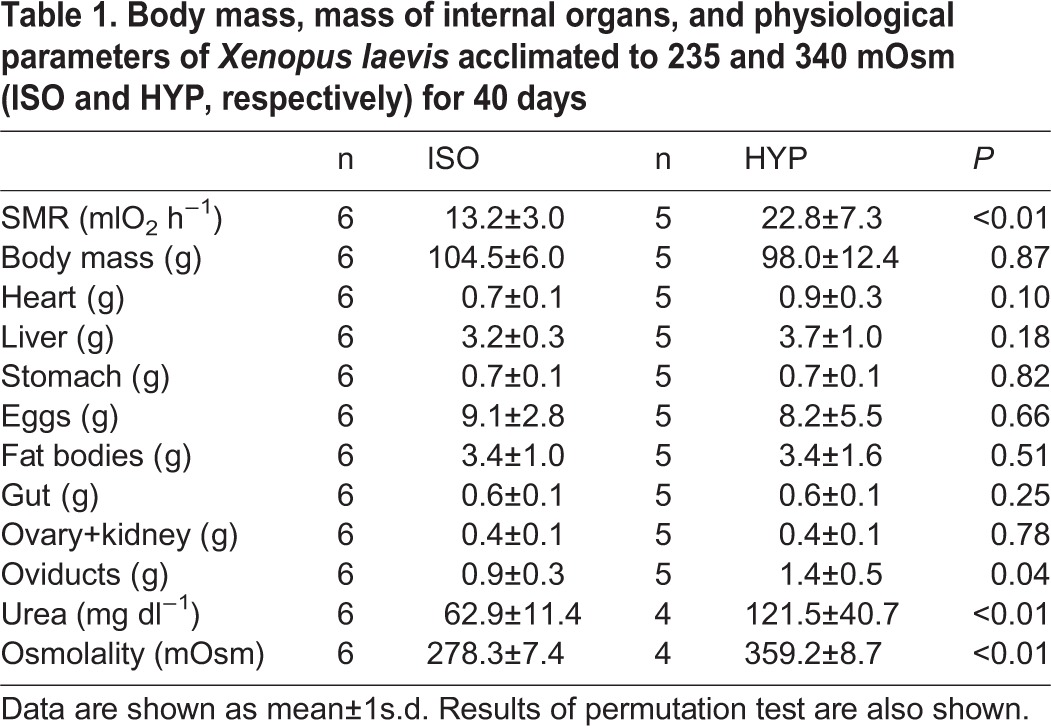
**Body mass, mass of internal organs, and physiological parameters of *Xenopus laevis* acclimated to 235 and 340 mOsm (ISO and HYP, respectively) for 40 days**

**Fig. 1.**
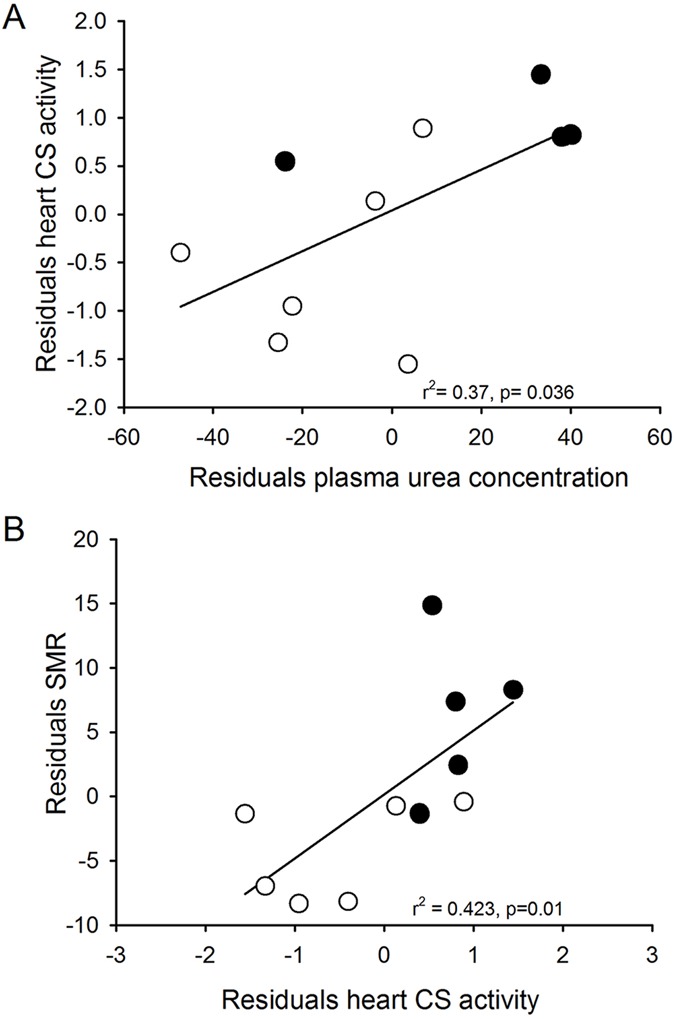
**Relationship between physiological parameters in *Xenopus laevis* acclimated to a medium with 235 mOsm (*n*=6, open circles) and 340 mOsm (*n*=5, closed circles), for 40 days.** (A) Correlation between residuals of citrate synthase activity of heart and residuals of plasma urea. (B) Correlation between residuals of citrate synthase activity of heart and residuals of standard metabolic rate. The *P*-value was obtained by permutation test (see text for details).

We found differences in the plasma urea concentration (mg dl^−1^) in the frogs of different treatments, with a higher value in the HYP group nearly 1.9 times that observed in the ISO group (Table 1). We observed non-significant relationships between the SMR with urea concentration in plasma, both in the conventional correlations (r^2^=0.32; Pt: *P*=0.082) and in the residuals analysis (r^2^=0.24; Pt: *P*=0.089). The residuals of urea concentration were associated positively with the residuals of heart (r^2^=0.470; Pt: *P*=0.010) and liver mass (r^2^=0.535; Pt: *P*=0.023). In the HYP group plasma osmolality was 1.3-fold higher than in the ISO group (Table 1). Furthermore, mean plasma osmolality of each group were slightly hyperosmotic in respect to the environmental water (Table 1). We also observed a positive association between residuals of plasma urea and residuals of citrate synthase activity of heart (r^2^=0.366; Pt: *P*=0.036; Fig. 1A).

### Organ masses

Mass of organs was unaffected by treatment after the acclimation period (see Table 1 for mean values and statistics). However, the analysis of organs corrected by body mass revealed a trend towards an increase of heart mass in frogs acclimated to 340 mOsm, both in the analysis by gram of body (Pt: *P*=0.029) and the residuals from mb (Pt: *P*=0.034). We found a positive relation between the plasma osmolality and the heart mass (r^2^=0.529; Pt *P*=0.027). The mass of the eggs was positively correlated with mb (r^2^=0.657; Pt: *P*=0.001).

### Enzyme activities

**Fig. 2.**
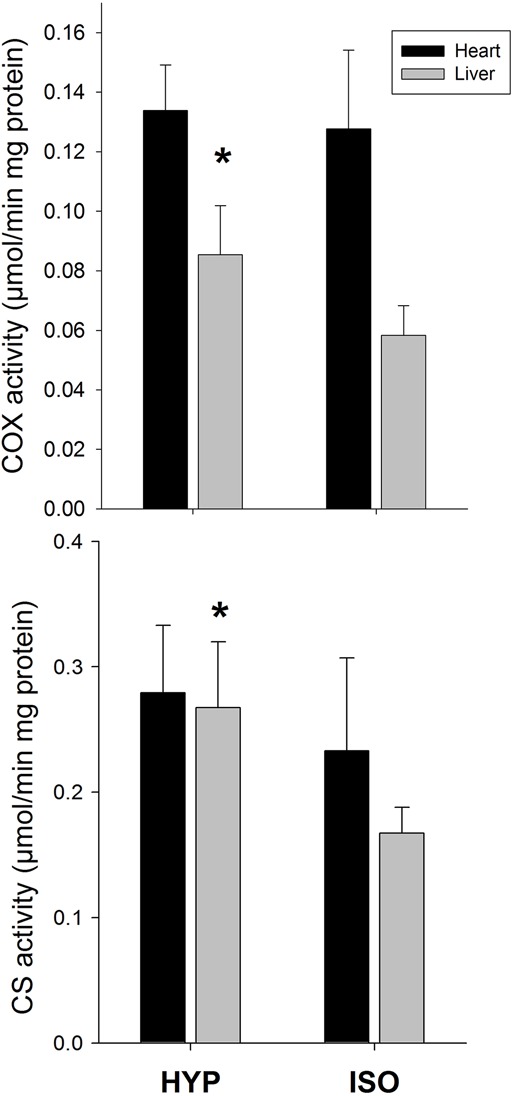
**Effects of salt acclimation on metabolic enzymes**. The activity of the enzyme citrate synthase and cytochrome c oxidase in liver of *Xenopus laevis* increased when frogs were acclimated for 40 days to an hyperosmotic medium (HYP, 340 mOsm, *n*=5) compared to frogs acclimated to an isosmotic environment (ISO, 235 mOsm, *n*=6). The enzyme activity was not affected by treatment in cardiac tissue. Data represented as mean±1 s.d.; *P<0.05.

Total citrate synthase activity in heart was significantly higher (200%) in the HYP group than in the ISO group (Pt: *P*=0.002, Fig. 2). However, total COX activity in heart was unaffected by treatment (Pt: *P*=0.602). The activity of CS and COX per gram of protein in the liver was affected by the treatment (*P*=0.008 and *P*=0.045, respectively; Fig. 2). Residuals of total CS activity in heart were positively correlated with residual of SMR (r^2^=0.423, *P*=0.042; Pt: 0.01; Fig. 1B). The activity of CS per mg of protein in liver presented a trend to be associated with the residuals of SMR (r^2^=0.349; *P*=0.072), however, the permutation test suggests that such relationship is significant (*P*=0.044).

## DISCUSSION

Similar to that found in other aquatic species, the long-term exposure of *X. laevis* to hypertonic environment elicited an osmoregulatory physiological response that lead to an increase in organismal rate of energy expenditure. We found that animals acclimated to hyperosmotic conditions elevated SMR by 173%. Moreover, metabolic variables were associated positively with osmoregulatory variables (Fig. 1). These results suggest the significant increase in energy expenditure of animals exposed to hyperosmotic conditions is associated with the increased osmoregulatory demand (e.g. urea and plasma concentration), and has the long-term effect of increasing the metabolic enzyme activities of associated organs, such as liver and heart.

In fact, *X. laevis* exposed for 40 days to a hypertonic solution of NaCl increased by around 20% total plasma osmolality and reached almost twice the urea concentration in plasma, compared with those exposed to an isosmotic condition. These results are in agreement with those reported previously (e.g. Ireland, 1973; Romspert, 1976; Katz and Hanke, 1993). Increased plasma urea concentration in amphibians is associated with a rise in the activity of the ornithine-urea cycle enzyme in liver, and urea retention in the kidney and urinary bladder (Balinsky, 1981; McBean and Goldstein, 1970). Such osmoregulatory responses may take a few hours to establish after transfer to the saline solution, along with the increase in plasma Na^+^ and Cl^−^ (McBean and Goldstein, 1970) and a decrease of Na^+^ transport by the skin (Balinsky, 1981). All of these mechanisms are thought to be energy demanding processes (see Uchiyama and Konno, 2006).

Coupled with increases in plasma urea and concentration, *X. laevis* exhibited higher specific activity of metabolic enzymes in liver and heart when acclimated to the hyper-saline treatment. This increase of CS and COX activity in liver suggest that the active synthesis of urea in response to chronic exposure to a hyperosmotic environment is accompanied by a greater metabolic activity of liver. In turn, the higher catabolic activity of heart in salt acclimated animals (approximate 200% increment, see Fig. 2) could be due to the higher need of oxygenated blood delivery to the liver because of its high tissue-specific rates of energy expenditure (Rieck et al., 1960). In fact, in the current study, increases in urea concentration can explain the increase in metabolic activity of heart of up to 37%. Interestingly, the increase in the activity of CS and COX may be explained by a higher mitochondrial density (Spinazzi et al., 2012) and/or by the production of a favored isozyme for the appropriate energy requirement. Thus, in a hyper-saline environment animals could synthetize a different enzyme, as it has been proposed, to occur in response to cold acclimation in amphibians (Berner and Bessay, 2006).

Thus, a close functional relationship between the metabolic rate and the catabolic capacity of organs associated with the osmoregulatory capacity (i.e. urea synthesis and oxygenated blood distribution) support our hypothesis about the presence of osmoregulation costs in a hyper-saline environment in amphibians. In this vein, studies developed in *Rana catesbeiana* revealed a positive association between the maximum aerobic capacity (by means of physical activity) and CS activity in heart (Walsberg et al., 1986). The authors suggested that the maximum aerobic capacity is limited in *X. laevis* by the ability of the cardiovascular system to deliver oxygen to the tissues. Accordingly, the observed increase in SMR was reflected in the metabolic changes of organs, particularly in the heart. In fact, variance in heart CS activity explains around 40% of the mass-specific SMR (Fig. 1B). Interestingly, overall increases in catabolic activity of a particular organ can be elicited by increments in specific activity, organ mass, or both (Kiss et al., 2009). In this case, *X. laevis* increased CS specific activity, but this strategy appears to be different to that found in other studies. For example, Gomes observed a positive relationship between liver masses and metabolic rates in genus *Scinax* (F. R. Gomes, PhD thesis, University of São Paulo, 2002; Gomes et al., 2004).

Despite our results showing a positive relationship between standard metabolic rate and plasma urea levels, this association seems to be dependent on the species and the ecological context in anurans. For instance, seasonal dormancy is commonly accompanied by hyperuremia in many amphibians from temperate regions (Muir et al., 2010). For example, studies evaluating the osmotic and metabolic responses to dehydration and urea-loading in a dormant frog (*Rana sylvatica*) found a negative correlation between SMR and plasma urea concentration (Muir et al., 2007). Thus, in *R. sylvatica*, urea could play a direct role in depressing metabolism of animals as a mechanism of saving limited energy reserves (Costanzo and Lee, 2005), affecting the structure and function of key regulatory enzymes (Muir et al., 2007) and promoting an hypometabolic state through the damage of proteins and enzymes (Muir et al., 2008). The same is true for *X. laevis*; it has been reported that this species can experience an increase in plasma urea in the presence of hyperosmotic environment (Katz and Hanke, 1993; Hochachka and Somero, 2002; Muir et al., 2007) but also in estivation or during migration (Balinsky et al., 1967; Malik and Storey, 2009), however, *X. laevis* did not exhibit an equivalent metabolic response in all these circumstances. Estivating *X. laevis* can reduce their metabolic rate along with increases in urea concentration (Hillman et al., 2009), developing a suppression of the activities of various metabolic enzymes (Merkle, 1989; Merkle and Hanke, 1988; Onishi et al., 2005) and recruiting anaerobic glycolysis (Katzenback et al., 2014). The differential response of *X. laevis* could be explained by the ability of this species to synthesize molecules that block the destabilization effect of urea on protein structure and function, such as trimethylamine (Wray and Wilkie, 1995). Therefore the increment of catabolic activity in liver and heart, along with urea concentration when exposed to hyperosmotic conditions, could be attained by this counteracting effect mechanism.

Thus, *X. laevis* exposed chronically to salt water incurred in metabolic cost in comparison to those exposed to isosmotic conditions. This supports the idea that the cost of osmoregulation in hyperosmotic environments is probably universal, regardless of osmoregulatory mechanism (Nehls, 1996; Morgan et al., 1997; Gutiérrez et al., 2011, 2012; Peña-Villalobos et al., 2013; Zikos et al., 2014). Furthermore, our study was not designed to investigate the metabolic cost of osmoregulation of *X. laevis* in a hypo-osmotic environment. It is very likely that *X. laevis* effectively requires expending energy by absorbing salts through the skin against a concentration gradient (Zerahn, 1956), however whether or not these putative costs are comparable to those incurred by the accumulation of urea in their tissues is an issue that requires further attention.

Finally, knowing the physiological responses of species to osmotic stress enhances our ability to understand the underlying mechanisms of biodiversity shifts driven by the current changes in the environment. Salinization of fresh water bodies constitute an acute form of environmental perturbation that has consequences on organismal fitness (Mack et al., 2000; Heine-Fuster et al., 2010). In freshwater ecosystems, increases in salinity have been shown to affect an organism's abundance (Amsinck et al., 2005; Sarma et al., 2006) and diversity (Jeppesen et al., 1994; Schallenberg et al., 2003). In this study, we analyzed the physiological consequences of the chronic exposition to salt water, and our data support the hypothesis that salinity may alter the energy budget in frogs by means of increasing rates of energy expenditure, likely related to the osmoregulatory costs associated with the synthesis and/or accumulation of urea in body fluids. Interestingly, the ability to produce urea as a response to osmotic conditions seems to be variable among anuran species. In fact, the capacity to concentrate urea in response to saline stress is higher in terrestrial or arboreal frog species than in an aquatic ranids (Grundy and Storey, 1994), probably as a consequence of differential ability to reabsorb urea by the kidneys and urinary bladder (Jørgensen, 1997). Thus, further studies are needed to evaluate to what extent the differences of urea accumulation imposed by hyperosmotic environments affect the mass-specific metabolic capabilities of amphibian species by means of acclimation. We suggest that the increased cost of maintenance produced by salt intake, as demonstrated in *X. laevis*, may significantly affect energy budgets, habitat tolerance and population survival in amphibians.

## METHODS AND MATERIALS

### Laboratory acclimation

Adult female *X. laevis* (*n*=12) were obtained from a feral population in San Antonio, a mesic coastal locality of central Chile (33834VS, 71836VW) characterized by a warmer summer and rainy and cold winters (mean annual precipitation 441.3 mm; Di Castri and Hajek, 1976). The watercourses where animals were trapped are characterized by having a variable salinity, with an average of 1.5 g l^−1^ NaCl (Vidal-Abarca et al., 2011). Animals were trapped in summer 2015, transported to the laboratory and randomly assigned to two water treatments (isosmotic, and saltwater) varying NaCl amount added to distilled water. Animals were maintained in individual plastics containers (28 liters) in a room at 25°C with a photoperiod light:dark 12 h:12 h. In the isosmotic group (ISO), the osmolality was increased at a rate of 23.13 mOsm/week, until 235 mOsm. This value was based on the plasma osmolality of *X. laevis* in freshwater environments (McBean and Goldstein, 1967). In the hyperosmotic group (thereafter the HYP group), the osmolality was increased weekly for two months from 50 to 340 mOsm, a NaCl concentration that is well tolerated by this species (Katz and Hanke, 1993), at a rate of 36.25 mOsm/week. Animals were fed twice a week with beef heart *ad libitum* for the two-month period required to reach the final experimental water concentration, attained by replacing water every two days. After reaching the expected concentration of NaCl, feeding was terminated (McBean and Goldstein, 1967; Katz and Hanke, 1993) and animals were acclimated for further 40 days at constant water salinity. Water was replaced each two days and the concentration was monitored by vapor pressure osmometry (Wescor 5130B). We used only adult females, because this allows us to evaluate the effect of salinity on egg quality, as a proxy for reproductive output. All experiments were conducted according to the Regulations for Animal Experimentation at University of Chile.

### Standard metabolic rate

After acclimation, standard metabolic rates (SMR) were estimated as the rate of oxygen consumption (VO_2_) using standard flow-through respirometry methods. SMR were measured during the photo phase period (between 10:00 and 14:00 h; Abel et al., 1992), using a protocol adapted from Kiss et al. (2009). Briefly, individuals were gently dried with a paper towel, weighed and placed in transparent acrylic chambers of 1.5 liters. This chamber was provided with a humid paper towel at the bottom and then located in a temperature controlled and illuminated cabinet (Sable Systems, Henderson, Nevada) at a constant ambient temperature (Ta=25±0.5°C). The metabolic chamber received air at 400 ml min^−1^ from a mass flow controller and through Bev-A-Line tubing (Thermoplastic Processes Inc., Georgetown, Delaware). The excurrent air passed through columns of Drierite, CO_2_-absorbent granules of Baralyme and Drierite before passing-through an Fox Box O_2_-analyzer equipped with a flow meter (Sable Systems) calibrated with a mix of oxygen (20%) and nitrogen (80%), which was certified by chromatography (BOC, Chile). The mass flow meter of the Fox Box was calibrated monthly with a volumetric (bubble) flow meter. The measurement and calibration protocols followed those of Williams and Tieleman (2000). Because water steam and CO_2_ were scrubbed before entering the O_2_ analyzer, oxygen consumption was calculated as: VO_2_=[FR×60×(Fi O_2_ – Fe O_2_)]/(1–Fi O_2_) (Withers, 1977), where FR is the flow rate in ml min^−1^, and the Fi and Fe are the fractional concentrations of O_2_ entering and leaving the metabolic chamber, respectively. Ten minutes of baseline O_2_ concentrations were recorded before and after each measurement period in order to correct for any drift in the O_2_ analyzer. Output from the oxygen analyzer (% O_2_) and flow meter was digitalized using a Universal Interface II (Sable Systems) and recorded on a personal computer using EXPEDATA data acquisition software (Sable Systems). Our sampling interval was 1 s. Frogs remained in the chamber for 4 h, long enough to reach steady-state conditions, which typically occurs after 1-2 h. We averaged O_2_ concentration of the excurrent airstream over a 20 min period after steady state was reached. After metabolic determinations, animals were sacrificed by cold exposure and decapitated (Katz and Hanke, 1993). Blood was collected in capillary tubes from heart, centrifuged at 12,000 ***g*** for 5 min, and the plasma was used to determine osmolality by vapor pressure osmometry (Wescor 5130B) and then stored (−80°C) for posterior urea analysis. Samples were thawed and the urea concentration (mg dl^−1^) was determined using the urease/Berthelot method with a commercial kit (Valtek, Chile). Samples were properly diluted to reach values that were within the range of the kit. Organs (liver, intestine, heart, ovaries plus kidney and stomach) and eggs were removed, and weighed (±0.001 g). Liver and heart were frozen at −80°C for further enzymatic determinations.

### Enzyme determinations

Tissues (liver and heart) were thawed, weighed and homogenized in 10 volumes of 0.1 M phosphate buffer with 2 mM EDTA (pH 7.3) with an Ultra Turrax homogenizer (20,000 rpm) (Janke & Kunkel, Germany) on ice. Samples were then sonicated at 130 watt for 20 s with 10 s intervals, 14 times each, using a Sonics Vibra-Cell VCX-130 (Conneticut, USA), maintaining the samples on ice. Cellular debris was removed by centrifugation for 15 min at 12,000 ***g*** at 4°C (Boeco M-240R, Germany). The supernatant was carefully transferred into a new tube, avoiding co-transference of the upper lipid layer present in the liver preparations. Protein concentration of the supernatant samples was determined using the method described by Bradford (1976), using bovine serum albumin as standard. Activities of two mitochondrial enzymes were determined: cytochrome c oxidase (COX; E.C. 1.9.3.1), and citrate synthase (CS; E.C. 4.1.3.7). COX activities were determined spectrophotometrically according to Moyes et al. (1997). Enzyme activity was determined in 10 mM Tris/HCl pH 7 containing 120 mM KCl, 250 mM sucrose, and cytochrome c reduced with dithiothereitol to a final volume of 0.2 ml. The decrease in optical density (O.D.) at 550 nm was monitored in 96-well plate spectrophotometer (Multiskan GO, Thermo Scientific, USA) at 25°C. Enzyme activity in units per gram of tissue was calculated using an extinction coefficient of 21.84 mM^−1^ cm^−1^ at 550 nm for cytochrome c. CS activities were measured according to Sidell et al. (1987). The CS assay medium contained 10 mM Tris/HCl, pH 8.0, 10 mM 5,5′dithiobis- (2·nitrobenzoic acid) (DTNB), 30 mM acetyl coenzyme A (acetyl CoA) and 10 mM oxaloacetic acid (omitted for the control) in a final volume of 0.2 ml. Citrate synthase catalyzes the reaction between acetyl CoA and oxaloacetic acid to form citric acid. The increase in O.D. at 412 nm was measured at 25°C. Enzyme activity was calculated using an extinction coefficient of 13.6 mM^−1^ cm^−1^ at 412 nm. We calculated mean specific activity as µmol min^−1^ per gram of protein. Because the residuals analyses required the value of total activities of the enzymes, we calculated the integrated activity of the whole organ as µmol min^−1^.

### Statistical analysis

Morphological, metabolic and biochemical data were compared between groups by means of a permutation test (Pt) of differences between groups with 10,000 permutations. We performed a permutation test in R, from the DAAG package; R Development Core Team (2009). For each treatment we evaluated the potential associations between physiological, morphological and biochemical variables by means of linear regressions, and performing permutation tests (Pt) for the coefficients of correlation (*n*=10,000). In cases when morphological and physiological variables were correlated with body mass (mb), we also used the residuals of those variables against body mass to perform the analysis. Statistical analyses were performed using the STATISTICA^®^ (2004) statistical package for Windows, and ‘R’ version 3.1.2. for Windows. Data are reported as mean±s.e.m. The numbers of cases for each variable may differ because some tissues were lost or damaged (e.g. plasma samples with hemolysis, were not employed in urea and osmometric analyses).
